# The Dynamic Family Home: a qualitative exploration of physical environmental influences on children’s sedentary behaviour and physical activity within the home space

**DOI:** 10.1186/s12966-014-0157-1

**Published:** 2014-12-24

**Authors:** Clover Maitland, Gareth Stratton, Sarah Foster, Rebecca Braham, Michael Rosenberg

**Affiliations:** School of Sport Science, Exercise and Health, University of Western Australia, 35 Stirling Highway, Crawley, WA 6009 Australia; Applied Sports Technology Exercise Medicine Research Centre, School of Engineering, Swansea University, Singleton Park, Swansea, SA2 8PP UK; Centre for Built Environment and Health, School of Earth & Environment and School of Sports Science, Exercise & Health, University of Western Australia, 35 Stirling Highway, Crawley, WA 6009 Australia

**Keywords:** Sedentary behaviour, Physical activity, Children, Environment, Housing, Family interview, Qualitative, Ecological model, Home space

## Abstract

**Background:**

Recent changes in home physical environments, such as decreasing outdoor space and increasing electronic media, may negatively affect health by facilitating sedentariness and reducing physical activity. As children spend much of their time at home they are particularly vulnerable. This study qualitatively explored family perceptions of physical environmental influences on sedentary behaviour and physical activity within the home space.

**Methods:**

Home based interviews were conducted with 28 families with children aged 9–13 years (total n = 74 individuals), living in Perth, Australia. Families were stratified by socioeconomic status and selected to provide variation in housing. Qualitative methods included a family interview, observation and home tour where families guided the researcher through their home, enabling discussion while in the physical home space. Audio recordings were transcribed verbatim and thematically analysed.

**Results:**

Emergent themes related to children’s sedentariness and physical activity included overall size, space and design of the home; allocation of home space; equipment within the home space; perceived safety of the home space; and the changing nature of the home space. Families reported that children’s activity options were limited when houses and yards were small. In larger homes, multiple indoor living rooms usually housed additional sedentary entertainment options, although parents reported that open plan home layouts could facilitate monitoring of children’s electronic media use. Most families reported changing the allocation and contents of their home space in response to changing priorities and circumstances.

**Conclusions:**

The physical home environment can enhance or limit opportunities for children’s sedentary behaviour and physical activity. However, the home space is a dynamic ecological setting that is amenable to change and is largely shaped by the family living within it, thus differentiating it from other settings. While size and space were considered important, how families prioritise the use of their home space and overcome the challenges posed by the physical environment may be of equal or greater importance in establishing supportive home environments. Further research is required to tease out how physical, social and individual factors interact within the family home space to influence children’s sedentary behaviour and physical activity at home.

**Electronic supplementary material:**

The online version of this article (doi:10.1186/s12966-014-0157-1) contains supplementary material, which is available to authorized users.

## Background

Participation in moderate to vigorous physical activity provides a range of health benefits in children and adolescents [1-3]. Conversely, time spent sedentary, in particular watching television (TV), has been associated with overweight and obesity, reduced fitness and poorer social and cognitive skills [4,5]. More recently, interruptions to sedentary time have been associated with lower waist circumference and better cardio-metabolic risk profiles in adults [6], although evidence is inconsistent in children [7,8]. Still, many children do not meet public health recommendations of at least 60 minutes of moderate to vigorous physical activity per day and less than two hours per day of sitting while using electronic media for entertainment [9-11].

Housing is a fundamental determinant of health and improvements in quality have been associated with better general, respiratory and mental health [12,13]. However, some changes within the home space, such as increasing electronic media and labour saving devices, have negative implications for public health as they facilitate sedentary behaviour and decrease opportunities for activity at home [14]. In Australia in 1998, just over 60% of households with children had access to a home computer and 20% had internet access at home. By 2008–09 this had risen to 91% and 86% respectively [15]. Additionally, from 1985 to 2009 the average new house size increased by approximately 50%, while private outdoor space, a location commonly used for children’s play, decreased [16-18]. Consequently, new houses with multiple indoor living areas designated for sedentary electronic media pursuits, such as TV viewing and computer use, are now commonplace. As children spend much of their time at home [19], they may be particularly vulnerable to the impact of changes within the home space.

Ecological models highlight the influence of environmental factors and recognise that behaviour is most likely shaped by the setting in which it occurs [20,21]. Ecological momentary assessment has shown that the majority of non-school sedentary behaviour occurs at home [22], and that time in the lounge room is most likely to be spent watching TV while time in the garden is most likely spent being active, highlighting the potential influence of location within the home space on behaviour [23]. Furthermore, electronic media equipment in the home and bedroom has been positively associated with electronic media use, a behaviour that most often occurs at home [24-26]. Yet, few studies have explored the relationship between children’s sedentary behaviours and the home physical environment, outside of equipment. Also, investigation of physical environmental influences within the home space and physical activity, has been largely limited to moderate to vigorous physical activity and active play outcomes across the entire day.

This study has chosen a qualitative approach, as research focusing specifically on physical environmental influences within the home space, and children’s sedentary behaviour and physical activity is lacking. Previous qualitative studies have noted yard space and sedentary entertainment options as barriers to active play and facilitators of electronic media use [27-29]. However, previous studies failed to investigate the physical environment of the home space in any depth and do not fully consider the context of children’s sedentary behaviours and physical activity. Therefore, the purpose of this study was to explore family perceptions of physical environmental influences on children’s sedentary behaviour and physical activity within the context of the home space.

## Methods

### Recruitment and participants

Participants were recruited to the HomeSPACE study via advertising through health units, non-government agencies and community groups. Families with at least one parent and one child aged 9 to 13 years, and living in the greater Perth metropolitan area, were eligible. Families registered their interest to participate via the study webpage, by providing their address and type of home, and selecting their perceived house and yard size from four options (small, medium, large or not applicable). Additional registrants were generated via snowball recruitment techniques.

The 50 families that registered were stratified into low, mid and high socio-economic status (SES) groups based on home location using the most recent version of The Australian Bureau of Statistics State Suburb Index of Relative Socio-economic Advantage and Disadvantage, 2006 [30]. Within each SES strata potential participants were selected based on self-reported house and yard size to ensure representation across study participants. Of the 30 families contacted, 29 verbally agreed to participate and were posted an information pack consisting of study details, consent forms and interview confirmation. After receiving the information pack one family chose not to participate leaving 28 families in the study. Thirty-seven percent, 73% and 100% of families living in high, medium and low SES suburbs respectively, who initially registered their interest to participate, were recruited to the study (Table 1). Active written consent was provided by parents and children. Each family was given a $30 retail voucher for their participation. The study was approved by the Institutional Human Research Ethics Committee.

**Table 1:** **Home characteristics of participant families - overall and by SES area group**

		**Overall**	**SES area group**		
		**(n = 28)**	**Low (n = 8)**	**Mid (n = 11)**	**High (n = 9)**
		**n**	**n**	**n**	**n**
House Size^a^	Small	5	1	3	1
	Medium	16	5	6	5
	Large	7	2	2	3
Yard Size^a^	No/Small	8	3	3	2
	Medium	14	4	7	3
	Large	6	1	1	4

^a^Self-reported.

### Procedure

The mosaic approach, as used by Clark (2010) to explore young children’s experiences in child care environments, is a framework that constructs meaning from a variety of active research methods with different individuals to compile an overall picture [31]. We used an adapted mosaic approach, that included a family interview [32], home tour [33] and observation, to elicit perspectives from both children and parents and obtain a comprehensive picture of the family home environment and the way the home space is used by children [34]. Insights gathered from interview locations can assist in the interpretation of qualitative research material [35]. Therefore, procedures were conducted in the family home so the interviewer could observe the physical space and interactions of family members with the space.

A semi-structured interview guide and background survey were developed and pilot tested with four families. The interview guide was based on a social-ecological framework [21], with an increased focus on physical home environmental factors compared to individual and social environmental factors. The background survey collected descriptive data on the participants including family demographics and the amount of portable and non-portable media equipment and physical activity equipment in the home space. Parents also reported the number of days in a usual week that their child was physically active for at least 60 minutes, and the time their child spent watching TV, playing video and computer games, and using the internet for leisure, on a typical week and weekend day. Questions were taken from a previous survey to assess home environments related to physical activity and sedentary behaviour [36] and adapted to the Australian context where appropriate. For example, for physical activity equipment a ‘trampoline’, which is common in Australia, was added and ‘snow equipment’ was removed.

The home visit comprised several parts (Additional file 1). First, to establish rapport, all participants were asked an introductory question about their favourite activity outside of school or work. Second, while parents completed the background survey, children were shown a series of 12 cards featuring common behaviours and then prompted to describe the three or four most common activities they do at home. Following this, children were asked to think of places within the home space where they spent the most waking time. Families then led the interviewer on a home tour discussing the physical elements of each space, typical activities that occurred in the space, family members who used the space and whether there were any rules for using the space. To complete the interview parents were asked additional questions about their home including how they chose their home and how it could be changed to increase physical activity and decrease sedentary time at home. Observations were recorded after leaving the interview under the headings house description, house layout, neighbourhood features and family characteristics.

As interviews included children and were conducted in family homes, the research process was somewhat flexible to deal with unexpected occurrences [32]. For example, the order of questions was changed if a child lost concentration or a parent attended to a phone call. On the occasion when parents asked that younger siblings be involved, the interviewer obliged but kept this to a minimum. The interview and tour were conducted in a range of spaces within the home from the family lounge room, to the dining table and the back yard. Detailed field notes on the house and yard, and equipment in each space, were taken after each interview. Interviews were conducted by the first author between June and November of 2012. Each interview lasted on average 69 minutes.

### Data analysis

Interviews were recorded using a digital recorder (Olympus WS-560 M), transcribed verbatim and anonymised. Any interruptions to the interview were noted and comments made by non-participating family members who were present in the home were removed. Thematic analysis using both deductive and inductive processes was used to analyse the transcripts [37]. Firstly, transcripts were read and notes on possible codes made by the first author. From this, a coding framework structured around the socio-ecological model was developed and verified by the study team. The data were imported into QSR NVivo10 (a qualitative data analysis software program) for coding and analysis. The data were initially coded into features of interest and then preliminary themes relevant to the research question were generated by the first author by collating codes into groups. Themes were reviewed, refined and then finalised by the study team. For further validation an external coder reviewed five randomly selected transcripts to verify the initial coding framework and substantiate the themes. Descriptive statistics of the sample were generated using IBM SPSS Statistics 19.

## Results

Seventy-four participants, including 41 children aged between 9 and 13 years, and 33 parents, participated in the 28 family interviews. The average age of the children was 10.9 years and 70.7% were male. Approximately one third (34.1%) of children participated in at least 60 minutes of physical activity a day in a usual week according to parental report. On a typical week and weekend day, 39.0% of children and 84.5% of children respectively, spent more than two hours a day watching TV, playing video and computer games, and using the internet for leisure. The majority of parents were female (78.8%) and two thirds had a university degree. Twenty-nine percent of families lived in low SES suburbs, 39% in mid SES suburbs and 32% in high SES suburbs. Most families lived in a separate home (85%). Self-reported home environmental characteristics of families are shown in Tables 1 and 2.

**Table 2:** **Equipment ownership of participant families - overall and by SES area group**

	**Overall**	**SES area group**		
	**(n = 28)**	**Low (n = 8)**	**Mid (n = 11)**	**High (n = 9)**
	**mean (range)**	**mean (range)**	**mean (range)**	**mean (range)**
Fixed electronic media^a^	6.8 (2–17)	7.0 (3–12)	6.2 (2–8)	7.2 (2–17)
Portable electronic media^b^	2.9 (0–8)	3.1 (1–8)	2.6 (0–6)	3.1 (1–5)
Physical activity equipment^c^	3.7 (1–6)	3.6 (1–5)	3.2 (1–5)	4.4 (2–6)

^a^Number of TVs, VCR/DVD players, desktop computers and fixed video game players in home.

^b^Number of handheld video game players, laptop and tablet computers accessible to child in home.

^c^Total of bike/scooter; sports, fixed play and gym equipment; swimming pool; and trampoline availability (max. score 6).

Five major themes relating to home physical environmental influences on children’s sedentary behaviour and physical activity were identified through analysis. Supporting quotes are provided to illustrate thematic findings, with participant type, and self-reported house size (LH - large, MH - medium, SH - small) and yard size (LY - large, MY - medium, SY - small, NY - no), following in brackets.

### Overall size, space and design of the home

Families perceived that the overall size, space and design of the family home were important influences on the sedentary behaviour and physical activity of children at home, particularly where space was limited. Private outdoor space was considered a requirement for physical activity at home by both parents and children. Although, having a larger sized yard was not always considered sufficient for children to use it for play.

*“I don’t have a chance to play outside as much because we don’t have the space to play outside…” (Boy, 10; SH, SY)*

*“Now, because it’s winter, and because we don’t have our dog anymore there’s not really much point to go out there.” (Boy, 13; MH, MY)*

Children’s available outdoor area was not always equal to the legal yard size. Some families viewed public land that abutted their block, particularly verges, as their territory and felt comfortable allowing children to play freely in these extended home space areas.

*“We’re pretty lucky we get an extra bit of verge. So it’s like an oval, so we play soccer out here and my son and I, we’ll kick the footy all the time…” (Male parent; MH, MY)*

While parents considered a yard with space for children’s play important when choosing their family home, location relative to schools, parks and other amenities was most frequently mentioned and a more important consideration.

*“Ideally we’d want somewhere with a bigger block where the kids could have a backyard and we could have a pool… We got into this area for high school.” (Female parent; MH, SY)*

Families reported that the design of the yard influenced the type of play opportunities available. Sport based play and running around required open space, while more creative free play was better facilitated by natural features.

*“It’s kind of a long space and there’s not many obstacles that you have to move around.”; “So it’s straight not wide, so it’s easier for your (football) accuracy.” (Brothers 10 & 12; MH, MY)*

*“Why is it a cool backyard?” (Interviewer); “Cause of that big tree over there. I really enjoy climbing it.” (Boy, 10; MH, MY)*

Families with smaller yards, or no yards, seemed to adapt in part to the lack of outdoor space by going out of the home for physical activity.

*“…we don’t really do a lot of activity in the home. I guess our life, maybe ‘cause we’re here, it’s structured that we do a lot outside of the home.” (Female parent; MH, SY)*

Many families had a large open plan area as the primary living space in their home. Generally parents and children reported preferring this design as it supported family interaction. Additionally, parents found that electronic media, particularly computers, could be housed in this communal area and their use more easily monitored. However, some parents commented that a television near the dining table in the open plan living area could entice children to watch during mealtimes or when other family members were watching.

*“That’s why we have the computer out there (open plan living area) ‘cause then I can see what they’re doing on the computer, but then most of the time we eat in here (dining room) away from the television.” (Female parent; LH, LY)*

A few parents noted that the availability and layout of indoor space combined with their supportive attitude made active play in the house more viable.

*“If they want to run inside the house, they know the rules… but the space is there so they’re not going to knock things over. I just wanted them to be able to play inside, kind of like they would outside.” (Female parent; MH, MY)*

### Allocation of the home space

Parents described allocating spaces in their home for specific purposes and dividing access among family members. Living spaces (areas other than the bedrooms, bathrooms and kitchen) were allocated to children or adults, or as communal family areas. Rooms were also given names to indicate their purpose, such as the study, games, theatre or craft room. Several families even had a ‘man’ space in the garage.

*“In the games room is all their computers, their Wii machine. That’s their pad. Their friends aren’t allowed in that lounge there. That’s the family lounge, so only when we go as a family we go into that room”. (Female parent; LH, MY)*

*“A man cave, that’s what we call it. Well we’ve got a foosball table and a couch, that kind of thing. Oh, all the sport equipment, stuff like that and dad’s building stuff…” (Boy, 11; MH, NY)*

Indoor spaces were generally viewed by parents as places for quiet play, including electronic media use, reading and creative activities. On the other hand, outdoor space was referred to by both parents and children as a place for the children to be active. Although children were aware of this view, many described participating in indoor active play at times.

*“If they want to go and play in their rooms they can play in their rooms but you don’t jump around. …if you want to play and run around, you go outside.” (Female parent; MH, MY)*

*“When it gets to going outdoors, we just use all our energy; and then we come back in and then rest for a while playing video games and stuff”. (Boy, 9; SH, MY)*

Many family homes had multiple living spaces that were primarily dedicated to sedentary electronic entertainment. Parents reported they preferred at least two distinct living areas, often so children and adults could watch TV separately. Some families also had a dedicated children’s games room for watching TV, playing electronic games (active and sedentary) and doing other activities such as playing with toys and drawing. Families in homes without multiple living areas found themselves competing for space.

*“That’s the whole purpose that the theatre’s there. We always wanted two living areas because the kids might want to watch one program and we might want to watch another.” (Parent female; MH, MY)*

*“Sometimes when my friends come over and my sister’s got a friend over and they’re watching a movie in the lounge room, we’ll watch a movie in my room.” (Boy, 9; SH, MY)*

*“If my father is working here, then we can’t play.” (Girl, 11; SH, NY)*

### Equipment, materials and furniture within the home space

Most families reported numerous electronic media devices including televisions, computers and electronic game consoles that provided a range of sedentary options.

*“When (my brother) gets the iPad, I usually play Wii or the computer. … if I get to the iPad, (my brother) might play Wii or the computer.” (Boy, 9; SH, SY)*

Parents felt computers were essential for children’s homework and education, and this made controlling their use for electronic games and social media more difficult. Portable electronic media provided additional challenges for monitoring, as it could be used anywhere within the home space.

*“Sometimes I just go on a game and then get back off it when Mum comes in the room.” (Boy, 12; MH, MY)*

*“The Wii’s here and he (my son) can’t escape … we all know where it is and what’s on it. Whereas his iPad can disappear from down here, upstairs quietly.” (Female parent; MH, NY)*

*“They are always “Ahh I’ve got to recharge it”, “Ahh I must keep it with me” … I’m going to have a cupboard with plugs; and everything, phones, laptops etc. go in there at night.” (Female parent; MH, MY)*

Parents indicated that active video games provided a better option than sedentary electronic games, but their use after purchase was inconsistent. However, children thought traditional electronic and computer games were better as they provided a greater range of games.

*“One of the reasons we did buy the Wii first was because it is a bit more active, and the Wii comes in and out of favour…” (Female parent; LH, LY)*

*“It (the computer) is so diverse. You can play so many different games on it rather than just the certain games that you actually have to buy.” (Boy, 12; SH, SY)*

Parents also reported providing equipment such as books and craft materials to encourage alternatives to electronic media use. In many families a large table was identified as the focal point for children to engage in projects, craft and other activities. This table was sometimes the communal dining table or a specific table set aside for these activities.

*“My mum hassled me and hassled me to read the first book and I got seriously into it that. …before I’d like desperately play with my iPad…” (Boy, 11; MH, NY)*

*“We do a lot on the table, like play games or if (my son’s) sorting stuff out or making stuff.” (Female parent; MH, MY)*

Sports and play equipment was frequently mentioned by children and parents as a facilitator of outdoor active play, with a pool, trampoline and basketball ring all popular. Children also enjoyed natural features, such as trees and grassed areas, for active play. Family pets facilitated active play and associated chores for children at home.

*“That’s one of the reasons why we put the pool in, so that they had an activity within the backyard that they could do unsupervised… rather than inviting friends over and everyone hanging around the TV screen.” (Female parent; MH, MY)*

*“There’s heaps of things to do (in the yard) like go in the spa, play basketball, jump on the trampoline and run around with (the dog).” (Boy, 12; MH, MY)*

When electronic media was not available, not working or not allowed to be used, children found other things to do such as playing outside or reading inside. However, with multiple electronic media devices available, some parents needed to restrict all devices before children chose an alternative.

*“We’ve had no DS’s, no computer games. We’ve had no broadband, we had the dialup. …(my son’s) not really that interested in all that.” (Female parent; LH, MY)*

*“Sometimes if I’m on my iPad and Dad says go and do something else, I go and watch the TV. And if Dad says don’t, they go off all together, sometimes I usually go up to my room and draw.” (Boy, 11; MH, NY)*

### Perceived safety of the home space

Safety was an issue raised by parents in relation to active play and electronic media use at home. Parents reported that elements of the home physical environment, such as fencing, lack of space and equipment location, could either raise safety concerns or alleviate them. Parents’ perceptions of children’s physical safety within their private outdoor space varied depending on the location and features of the yard. Backyards were considered a safe place for children to play. Playing in front yards, near busy roads with inadequate fencing, was seen as dangerous. In homes where parents could see the front yard from inside the house, there was a large verge, or the home was on a quiet street, there were fewer safety concerns and play was more likely to be allowed.

*“…we play outside, but we have to play with our parents because there’s no safety outside.” (Girl, 11; SH, NY)*

*“They can play in the back yard… but in the front yard you’ve got cars going up and down the road, you’ve got people going up and down the road.” (Female parent; MH, MY)*

Parents also often restricted play inside the house due to concerns about injury from falling or running into objects, as well as breaking household items. Some parents in more spacious homes did not seem to perceive the same level of risk.

*“I send them outside, just out of fear of them belting their heads or going through the glass windows…” (Female parent; MH, LY*)

Cyber safety was mentioned by many parents as an important issue surrounding children’s electronic media use. Locating the computer in an open communal area where children could be monitored was a preferred strategy for many parents to enhance safety.

*“It’s also always been a rule that the computer faces where people can walk past so you just can’t hide anything.” (Female parent; MH, MY)*

### The changing nature of the home space

Families revealed that the home environment is constantly changing. Many families reported they had recently made or were planning to make major changes, such as moving homes or renovating their current home to increase functionality and space. Parents and children noted that these changes influenced the types of activities that were available to do at home.

*“Now I can just kick the soccer ball on the wall. But at our old house there was no wall just a fence…” (Boy, 10; MH, MY)*

Almost all families discussed smaller changes such as purchasing equipment that enabled a specific sedentary behaviour or activity. Examples included an ipad for homework, an electronic gaming console for playing electronic games and a trampoline for outdoor play.

*“With the new Xbox, he’s been on it a bit. Usually when he doesn’t have it, he’s outside.” (Male parent; LH, LY)*

Modifying the home environment seemed to be driven either by changes within the family, such as family structure and children aging, or by the preferences and priorities of parents and children.

*“You go through the stage where you’ve got the totem pole and the swings and the trampoline and then as they get older, they move out and then in comes the swimming pool…” (Female parent; MH, MY)*

*“The boys particularly spend the whole time pretty much outside and so I like having lots of space for them and that was a big consideration for the renovation to keep space outside.” (Female parent; LH, LY)*

For families on lower incomes, who lived in apartments or who rented, overall home size, space and layout were not readily changeable. However, families discussed how they were able to at least partially adapt to the structural limitations of their home. For example, families with a smaller yard reported going to the park more often. One family allowed cricket to be played indoors as there was no enclosed yard and another family moved furniture around whenever they wanted to play active video games.

*“So walking to the park and everything like that, that’s become our way of life, because we don’t have the space at home to mess around in the backyard.” (Female parent; MH, SY)*

*“We moved it (coffee table) here and then Mum kept saying that it was in the way of the walkway so we had to move it in front of the couch (to play Wii).” (Girl, 11; MH, MY)*

## Discussion

**Figure 1:**
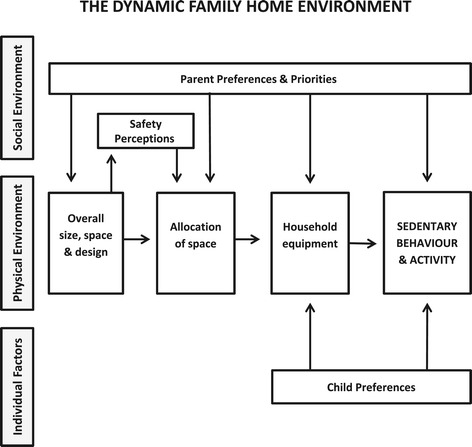
**Summary of family perceptions of physical environmental influences on children’s sedentary behaviour and physical activity within the home space in a social ecological framework.**

The study findings indicate that families perceive the physical environment of the home space influences children’s sedentary behaviour and physical activity via: overall size, space and design of the home; allocation of home space; equipment within the home space; and perceived safety of the home space. Furthermore, the home space seems to be a dynamic environment where many of the physical elements are chosen, controlled and changed by family members, particularly parents. Accordingly, social environmental and individual factors may influence children’s sedentary behaviour and physical activity at home both directly and through the creation of the home physical environment. Figure 1 summarises the study findings.

Findings suggest that elements of the home physical environment frequently change, and these changes are largely affected by the family living within the home. We found parents’ preferences and priorities, influenced by their children, were most important in the series of decisions that shape the physical environment of the family home: selecting the overall home, allocating space and providing equipment. As family circumstances changed and children matured, preferences and priorities altered, and consequently the home physical environment was modified or changed. A previous review of building design and physical activity describes similar stages in the creation of office environments, and consistent with our findings, found that each of these stages can affect physical activity, but have different decision makers and rates of change [38]. However, unlike other environmental settings that are not directly under the control of individuals, the current study suggests a high degree of individual control over the home space and its use. These observations are consistent with the Social Cognitive Theory concept of reciprocal determinism, that recognises that while the environment can facilitate or act as a barrier to behaviour, individuals can construct environments to suit themselves [39]. When asked about changing the home physical environment to increase activity or decrease sedentariness, parents reported both changing features and equipment in the physical environment, and controlling how home space and equipment are used. This ability for individuals within the home to directly control major elements of the physical environment and capacity for change seems to differentiate the home from other settings such as schools and neighbourhoods.

### Indoor space

Our findings indicate that the trend towards larger houses and more indoor living space provides families with more options when allocating space for people and purposes. Additional living space allowed children their own lounge or games room which could potentially be used for sedentary behaviour or activity. However, we found most indoor living spaces were designated for sedentary leisure with a range of electronic media options. Rather than increasing activity these additional living spaces decreased competition between family members and provided more autonomy in the choice of sedentary pursuits. Autonomy is a key driver of behavioural motivation in Self-Determination Theory [40], and allocating indoor space to a child may increase autonomy and sense of control within the space, thereby reinforcing the predominantly sedentary behaviours that occur there.

Open plan living areas were a focal point in the family home where a range of sedentary behaviour, activity and social interaction occurred together in the same space. Electronic media rules have been associated with less TV time [41], and placing the TV and computer in an open plan area enabled some parents to better monitor media use and enforce rules. However, depending on the physical layout and how furniture was configured, the TV could potentially be seen from the dining table, which is of concern as the number meals taken while watching TV has been positively associated with increases in children’s TV time [42]. In human geography, the open plan living area has been described as a space that can accommodate a range of family practices and be physically configured to suit the family living within it [33]. This concurs with our findings that the final make-up of the open plan living area, and the home physical environment generally, is largely shaped by the family, particularly the parents, who choose to allocate space and place equipment and furniture within the house.

Many parents viewed indoors as a space for quiet play, such as electronic media use and reading, and outdoors as a space for active play. Children also stated this view, although did not always adhere to it. The home physical environment mostly reinforced this ideal through children’s ‘lounge’, ‘games’ or ‘activity’ rooms that included a TV and electronic games, as well as items such as toys and books. Recent qualitative research also found that children’s play now encompasses both active play and indoor sedentary play such as electronic gaming [43]. Parents that allowed active play inside attributed this to having indoor space available, as well as supporting or tolerating more boisterous activities inside. The exception was active video gaming, where parents did not have the same injury or breakage concerns as for other less controlled forms of active indoor play. Laboratory studies have found active video gaming can increase energy expenditure and improve vascular function [44,45], although home based active video gaming interventions have been less successful in increasing physical activity [46]. One intervention conducted in The Netherlands found lack of home space was an issue for active video gaming [47]. However in our study most families with more than one living area had no problem in finding a permanent space for active video gaming. Together, these findings suggest that with a moderate amount of indoor space, children can engage in active indoor play at home. However, the physical environment, parental safety concerns and family rules, all interrelate to limit or support children’s activity indoors.

### Outdoor space

Families in this study reported that having a small or no yard limited active play and increased sedentariness, as children were more likely to be indoors when at home. Yet, despite similar findings in previous qualitative studies [29,48], there is only limited evidence of any association between yard size and children’s outdoor play in observational studies [49,50]. Our findings showed that families with limited outdoor space often adapted their behaviour by taking children outside the home for structured activity or to the park, which may explain this apparent inconsistency. We also found yard size did not always equate to space available for play as many families allocated an area for outdoor dining in their yard. Additionally, while parents have reported the yard as the safest place for active play [18], some parents in this study perceived the front yard as unsafe for play due to inadequate fencing, busy roads and uncivil neighbours. Concurring with this, an Australian urban planning study found distribution of yard around the house can diminish usable outdoor space [51]. Furthermore, we found children also needed a reason to go into the yard, such as physical activity equipment, a preference for activity or family members to play with. Hence, while the size of private outdoor space may limit or facilitate active play at home, yard space may be less crucial for children in families who have the capacity to support other opportunities for active play and physical activity.

### Electronic media

The pervasiveness of electronic media in the family home has a major impact upon the how the home is used by children. TVs, desktop computers and electronic game consoles can be placed in relatively fixed locations within the home, while laptop, tablet computers and handheld devices can feasibly be used almost anywhere. Having a TV and other electronic media in the bedroom has been associated with more sedentary electronic media use [24,26] and less reading [52]. In our study, a range of living areas, including the open plan living area, lounge and games room were used for screen based media, with use in bedrooms less prominent. However, research from the UK, where the average urban house size is considerably smaller [53], found children’s screen based leisure mostly occurs in the bedroom, as well as the main living area of the home [54]. This possibility that smaller overall living space may decrease the options for accommodating electronic media is worthy of further consideration, as it may be a barrier to removal from children’s bedrooms.

Parents in this study reported that children’s use of multifunctional and portable electronic media was challenging to control, as these devices could be used anywhere and for a range of educational, entertainment and social purposes. An earlier Australian study found that parents who were concerned about children’s TV viewing had fewer TVs and less electronic media in the home, and placed more restrictions on electronic media use [55]. This is consistent with our findings that some parents deliberately delayed or limited the purchase of electronic media to restrict use and many parents had rules around where, when and how devices could be used. Hence, strategies put in place by parents, such as limiting electronic media purchases, housing portable electronic media outside the bedroom and electronic media rules, seem to be most important in controlling the use of ubiquitous multifunctional and portable electronic media.

### Future research directions

Research on the home physical environment to date has centred on establishing correlates of physical activity and electronic media use. There has been little investigation of potential home physical environmental influences, outside of equipment, and of how home physical and social environmental factors interact [56]. These findings highlight additional influences perceived by families on children’s sedentary behaviour and activity at home including house and yard size, space and design, and placement of equipment and furniture. More comprehensive measures of home physical environments that incorporate these influences and the location of equipment are required for use in observational studies. Reviews of the built environment and physical activity recommend that physical environmental influences be investigated as moderators of the relationship between social and individual factors, and physical activity [57]. Exploration of these relationships within the home environment is also lacking [56], with the findings of this study indicating that parental factors including preferences, priorities and rules, may influence children’s behaviour indirectly through the structuring of the home physical environment. Interventions that change the environment in small ways, such as placing computers in open areas, removing electronic media from bedrooms and allocating areas for play space, are yet to be assessed for efficacy in this context. Also, further exploration of the role of new generation and portable media, including smart phones, tablet computers and electronic gaming devices, in facilitating sedentary behaviour, and potentially activity, will be an important research direction, particularly as technology becomes woven into family homes.

### Strengths and limitations

This study is unique by virtue of its thorough exploration of the physical environment in which children’s sedentary behaviour and physical activity occurs at home. A major strength of the study was the research design. The family interview and home tour allowed the interviewer to observe the physical environment and the interactions of participants with the home space. The observations and tour, in addition to the opinions of both parents and children, provided a more comprehensive picture of sedentary behaviour and physical activity in the family home, than might have been provided by only one method.

However, research results should be considered in light of several limitations. Firstly, participants all resided in the Perth metropolitan area and findings may not be generalizable to families and homes in other regions. It should be noted that the average Australian house size is one of the largest in the world [16] and this may have influenced participants’ perceptions of house and yard size. Secondly, participating families were selected from low, mid and high SES suburbs in an attempt to ensure equal representation. However, tertiary educated parents were overrepresented. In children, two thirds of participants were boys, and it should be noted there are gender differences between the behaviours of boys and girls at this age [58,59]. This may indicate that families who registered to participate in the study felt that home space was more pertinent for boys. And finally, it should be also acknowledged that answers, particularly those given by children in the presence of parents, may be prone to social desirability.

## Conclusions

The findings of this study suggest that overall size, space and design of the home, and the allocation of space and equipment within the overall home space, can influence the sedentary behaviour and physical activity of children at home. Additionally, many elements within the family home space are manipulated to suit the preferences and priorities of the family living in the home. This ability to create, control and change the physical environment of the home space seems to differentiate the home from other environmental settings where individuals have less control over their environment. While families considered physical environmental factors important, how families use their home space and overcome challenges posed by the environment also appears important for limiting children’s sedentary behaviour and increasing physical activity at home. We propose that further scientific endeavour should investigate the direct effect of the home physical environment on children’s sedentary behaviour and physical activity at home and seek to understand how parental factors, including preferences and priorities, influence the formation of home physical environment.

## Additional file

Additional file 1:
**The HomeSPACE Study Family Discussion Guide.**
