# Supplementary material for: The Dynamic Family Home: a qualitative exploration of physical environmental influences on children’s sedentary behaviour and physical activity within the home space
Source: Int J Behav Nutr Phys Act. 2014 Dec 24;11:157. doi: 10.1186/s12966-014-0157-1 (PMC4304138; doi:10.1186/s12966-014-0157-1)
Supplement: Additional file 1: — The HomeSPACE Study Family Discussion Guide. [file 12966_2014_157_MOESM1_ESM.pdf]

Participant:

Date:

The HomeSPACE Study

Family Discussion Guide

**Introduction (5-10 mins)**
I'm _______________ from the School of Sport Science, Exercise and Health at UWA. Thanks for agreeing and making time to take part in an interview. Is there a place we can sit down to talk?
**Ethics Forms**<br>Before we start I need to confirm that you received all of the information about the study including the consent forms. Did you have any questions about the information you have been given or the consent forms? Do you have those with you? Are you happy to sign those? I will take these with me and send you a copy for your records.<br><br>**Gift Voucher**<br>This is your gift voucher as a thank you for your time today. I'll give this to you now so I don't forget. I need you to sign off on receiving it. Could you please write your name and sign the form.<br><br>**Format**<br>Today I'll be asking questions about how you use your home for different activities - both activities that involve moving and activities that involve sitting. Everything you say is really interesting and important to me. There are no right or wrong answers.<br><br>**Tape Recording**<br>Because we'll be discussing a lot of different things today, for me to remember everything I would like to audio record our discussion on this digital recorder. All information collected today, including this recording, will be strictly confidential. All the information is kept secure and no one will be identified by their name from this research. Your participation is voluntary and you may withdraw from the discussion at any time for any reason.<br><br>Are you are you okay with me starting the audio recording and the interview?<br><br>Can you please say your name, age and favourite thing to do outside of school/work hours. This will help me to recognise your voice on the recorder.

Survey / Card Sort (10-15 mins)

I'll start by asking Mum/Dad to complete a background survey and while they are doing that we'll do a short activity with some cards and talk about them. How does that sound?

Survey (Parent only)
Here is a brief background survey to complete. This should take about 10 minutes. The survey is very important as it provides us with some background information about you, your house and your family. Try to answer all the questions the best you can. Is that okay?

Usual Activities - Card Sort Activity (Children only)
While you're completing that I'm going to ask ____________ to talk about what they do on a usual weekday. Is that okay for both of you?

There's a pack of cards in front of you - take a look at the cards and choose the 3 activities that you do most on a typical school day outside of school hours – so before school, straight after school and evening. Then we'll talk about them. *Prompt: Start in the morning before school, after school, evening. Think about yesterday.*
Which activity do you do most? Where do you do this? Do you do with others or by yourself? *Do for all activities.* Which do you like best?

And what about a usual weekend day? Take the cards and select 3 cards based on a usual weekend day. *Prompt: Start in the morning, through the day, and evening? Think about last Saturday.*
Which activity do you do most? Where do you do this? Do you do with others or by yourself? *Do for all activities.* Which do you like best?

Usual Activities (Child only)
What are your favourite things to do as a family when you are at home? Where do you do this?
Prompt: Think about a Sunday evening?
What are your favourite things to do by yourself when you are at home? Where do you do this?
Prompt: is it different to what you do together?
What about when friends come over? What things do you do at home? Where do you do this?
Prompt: is it different to what you do by yourself?

Indoors vs Outdoors (Family)
Some people describe themselves are being 'outdoorsy' or "indoorsy' people. How would you describe yourself? *Prompt: Would you say you are more indoorsy or outdoorsy? Do you like being active like running around or playing sport in your spare time? Or doing things like watching TV, being on the computer or reading?*
Why do you say that? What makes you choose to do something active like playing in the yard vs watching TV or playing on the computer?

Home Tour (20-25 mins)

Spaces for Activities (Family)
Now I'd like to talk with you about the different places in your house and yard that you use for different activities. I'd like to do this as a moving tour. What that means is I'll ask ________ to think of places in your house and yard where he/she spends the most time. Then we'll go to those places and you can tell me all about the place and how you use that place. It's completely up to you where the tour goes. You are in charge. And we can come back here at any time. Does that sound okay?

_________, think of the three places in the house and yard where you spend the most time when you are awake. What are they? What about we start in the ___________ room?

For Each Room… (Family)
Tell me about this area/room?
What types of activities happen in here?
How much time do you spend in here?
Who are you usually with?
What equipment do you have in here (*prompt: media, play equipment, seating*)?
Is it easy to get to the equipment you have?
Is there enough space to do what you would like to?
Do you have any rules for what happens in here?
If you could change this room in any way what would you do?

For Video Games Consoles (as appropriate)…
I noticed you've got video game player.
What sort of games do you play on there? Tell me about why you like those games?
Are they movement based or regular games? Who do you play with?
Do you have enough space to play?
Are there any rules around playing? Are the rules different for movement based vs regular games?
What makes you choose to play a movement based game over a regular one?

After 3 rooms…
Is there anywhere else you'd like to show me? If not lets go back to where we started.

Final Questions (15-20 mins)
**Spaces for Activities (Family)**<br>Thinking about your home. What are your favourite features of the house and yard?<br>What do you most like and why? What do you most dislike and why?<br><br>Do you have access to mobile technology like laptop, smart phone, handheld electronic game etc? Where do you usually use these at home? Where do you usually access the internet at home?<br><br>That was my last question for you both about the activities that you do in your home. I have a few more questions for Mum/Dad so ____________ you can either stay with us or if Mum/Dad is happy you can go. Is there anything else you would like to add?<br><br>**Housing (Parent Only)**<br>Why did you choose this home for your family to live in? What things did you consider?<br>If you could change anything about your home what would it be? Why would you do this?<br><br>What if you could redesign your house to do things like rearrange the rooms or make areas bigger or smaller? What would you do?<br>What if you had to make changes to the house & yard to make it more active? What would you do?<br>What if you had to make changes to the house & yard to get the kids away from screens and spend less time on the couch? What would you do?<br><br>**Rules (Parent Only)**<br>Do you have any rules around media use we haven't already spoken about? Tell me about these.<br>*Prompt: When? Where? How long? Do these apply to the whole family?*<br>Do you have any rules around playing inside or outside we haven't already spoken about? Tell me about these. *Prompt: When? Where? How long? Do these apply to the whole family?*
**Conclusion (5 mins)**
**Last Questions**<br>Is there anything else you'd like to add? *Prompt: Anything to add to what we have talked about?*<br><br>**Additional Recruitment**<br>We are looking to interview 30 families for this study. Is there anyone else you know who may be interested in participating? How about I email you with the study information which you can pass on? We can provide them with information and the opportunity to register should they want to.<br><br>**Thank You & Goodbyes**<br>Thank you so much for allowing me to come and interview you and your family. Your time and your views are greatly appreciated. All the information you've provided will be very helpful in shaping future research, policy and programs in the area of children's activity, housing and health.

Field Notes

House Description

House Layout

Field Notes

Neighbourhood Features

Family Characteristics

Additional Notes
